# Seasonal dynamics, *Leishmania* diversity, and nanopore-based metabarcoding of blood meal origins in *Culicoides* spp. in the newly emerging focus of leishmaniasis in Northern Thailand

**DOI:** 10.1186/s13071-024-06487-z

**Published:** 2024-09-19

**Authors:** Chulaluk Promrangsee, Sira Sriswasdi, Sakone Sunantaraporn, Chatuthanai Savigamin, Thanapat Pataradool, Chatchapon Sricharoensuk, Rungfar Boonserm, Rinnara Ampol, Pitchayaporn Pruenglampoo, Mathirut Mungthin, Jonas Schmidt-Chanasit, Padet Siriyasatien, Kanok Preativatanyou

**Affiliations:** 1 Interdisciplinary Program of Biomedical Sciences, Graduate School, Chulalongkorn University, Bangkok, Thailand; 2 Center of Excellence in Computational Molecular Biology, Faculty of Medicine, Chulalongkorn University, Bangkok, Thailand; 3 Center for Artificial Intelligence in Medicine, Research Affairs, Faculty of Medicine, Chulalongkorn University, Bangkok, Thailand; 4 Department of Integrated Biosciences, Graduate School of Frontier Sciences, The University of Tokyo, Kashiwa, Chiba Japan; 5 Center of Excellence in Vector Biology and Vector-Borne Disease, Chulalongkorn University, Bangkok, Thailand; 6 Department of Parasitology, Faculty of Medicine, Chulalongkorn University, Bangkok, Thailand; 7 Division of Medical Technical and Academic Affairs, Department of Medical Services, Ministry of Public Health, Nonthaburi, Thailand; 8 Department of Parasitology, Phramongkutklao College of Medicine, Bangkok, Thailand; 9 Bernhard-Nocht-Institute for Tropical Medicine, Bernhard-Nocht-Str. 74, Hamburg, Germany; 10 Faculty of Mathematics, Informatics and Natural Sciences, Universität Hamburg, Hamburg, Germany

**Keywords:** *Culicoides* biting midges, *Leishmania*, *Mundinia*, Nanopore metabarcoding, Host preference, Killick-Kendrick’s criteria, Northern Thailand

## Abstract

**Background:**

Clinical cases of leishmaniasis caused by *Leishmania* (*Mundinia*) parasites have been increasingly reported in Southeast Asia, particularly Thailand. Recent evidence has shown that *Leishmania* (*Mundinia*) parasites successfully developed into infective metacyclic promastigotes in *Culicoides* biting midges, strongly supporting their putative role in disease transmission. However, *Culicoides* diversity, host preference, and *Leishmania* prevalence in endemic areas remain largely unknown.

**Methods:**

We investigated the seasonal dynamics, infection prevalence, and blood meal identification of *Culicoides* collected from the emerging focus of visceral leishmaniasis in Lampang Province, Northern Thailand, during 2021–2023. Midge samples were molecularly screened for *Leishmania* using *SSU rRNA*-qPCR and *ITS1*-PCR, followed by Sanger plasmid sequencing, and parasite haplotype diversity was analyzed. Host blood meal origins were comparatively identified using host-specific *Cytb*-PCRs and a nanopore-based metabarcoding approach.

**Results:**

A total of 501 parous and gravid females and 46 blood-engorged ones belonging to at least 17 species of five subgenera (*Remmia*, *Trithecoides*, *Avaritia*, *Hoffmania*, and *Meijerehelea*) and two species groups (*Shortti* and *Calvipalpis*) were collected with temporal differences in abundance. *Leishmania* was detected by *SSU rRNA*-qPCR in 31 samples of at least 11 midge species, consisting of *Culicoides oxystoma*, *C. guttifer*, *C. orientalis*, *C. mahasarakhamense*, *C* (*Trithecoides*) spp., *C. innoxius*, *C. shortti*, *C. arakawae*, *C. sumatrae, C. actoni*, and *C. fulvus*, with the overall infection prevalence of 5.7%. The latter six species represent the new records as putative leishmaniasis vectors in Northern Thailand. The *ITS1*-PCR and plasmid sequencing revealed that *Leishmania martiniquensis* was predominantly identified in all qPCR-positive species, whereas *L. orientalis* was identified only in three *C. oxystoma* samples. The most dominant haplotype of *L. martiniquensis* in Thailand was genetically intermixed with those from other geographical regions, confirming its globalization. Neutrality test statistics were also significantly negative on regional and country-wide scales, suggesting rapid population expansion or selective sweeps. Nanopore-based blood meal analysis revealed that most *Culicoides* species are mammalophilic, with peridomestic and wild mammals (cow, pig, deer, and goat-like species) and humans as hosts, while *C. guttifer* and *C. mahasarakhamense* fed preferentially on chickens.

**Conclusions:**

This study revealed seasonal dynamics and sympatric circulation of *L. martiniquensis* and *L. orientalis* in different species of *Culicoides*. Evidence of human blood feeding was also demonstrated, implicating *Culicoides* as putative vectors of human leishmaniasis in endemic areas. Further research is therefore urgently needed to develop vector control strategies and assess the infection status of their reservoir hosts to effectively minimize disease transmission.

**Graphical Abstract:**

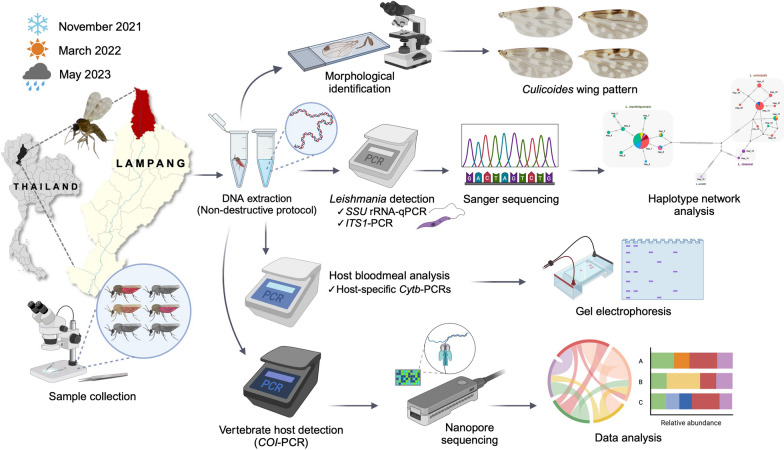

**Supplementary Information:**

The online version contains supplementary material available at 10.1186/s13071-024-06487-z.

## Background

Autochthonous leishmaniasis is currently considered an important public health problem in Southeast Asia with Thailand being an endemic hotspot. Since 1996, an increasing number of clinical cases have been reported, particularly in the northern and southern provinces of the country [1]. This emerging disease has been proven to be caused by two *Leishmania* species of the newly classified subgenus *Mundinia*, namely *Leishmania martiniquensis* and *L. orientalis* [1–5]. Traditionally, leishmaniasis was known to be transmitted by phlebotomine sand flies [6]. Based on the positive detection of *Leishmania* DNA, several phlebotomine species of the genera *Sergentomyia* [7–9], *Grassomyia* [10], and *Phlebotomus* [11] were proposed as potential vectors of these two *Leishmania* (*Mundinia*) species. However, recent evidence indicates that several *Leishmania* (*Mundinia*) parasites could not successfully develop to the infective stage within the midgut of the sand fly [12–14]. In addition, the positive detection rates for *Leishmania* (*Mundinia*) species are relatively low in field-caught sand flies, suggesting that these emerging *Leishmania* (*Mundinia*) parasites may be transmitted by other blood-sucking dipteran vectors than sand flies [7, 9–11, 15].

In addition to sand flies, *Culicoides* biting midges are globally distributed hematophagous insects of the family Ceratopogonidae, order Diptera. Of medical and veterinary importance, species of this insect genus have been implicated as vectors of several human, domestic, and wildlife pathogens. They are involved in the transmission of several animal arboviruses, including African horse sickness, bluetongue, epizootic hemorrhagic disease, Oropouche, vesicular stomatitis, and Schmallenberg viruses [16]. *Culicoides* species have also been incriminated as vectors of protozoan parasites infecting mammals and birds, including *Hepatocystis*, *Haemoproteus*, *Leucocytozoon*, and *Trypanosoma*, as well as several species of filarial nematodes [17–20].

It has been previously noted that several *Leishmania* species, including *Leishmania* (*Leishmania*) *amazonensis* [21], *L.* (*L.*) *infantum* [22], *L.* (*L.*) *mexicana* [23], and *L.* (*Viannia*) *braziliensis* [21], have been molecularly detected in *Culicoides* spp. collected from different geographical origins. Nevertheless, the metacyclic development of these *Leishmania* species has never been demonstrated in dissections of natural *Culicoides* species or in laboratory experiments, which is an important criterion for confirming their vector competence [12–14, 24, 25]. However, recent experimental infections have shown that several strains of *L. martiniquensis* and *L. orientalis* can complete metacyclic development in *Culicoides sonorensis* and be successfully transmitted to mice [12, 14]. It is therefore highly likely that *Culicoides* biting midges play a role in the transmission of autochthonous leishmaniasis caused by these two *Leishmania* (*Mundinia*) species in endemic areas of Thailand.

Positive detection of two autochthonous *Leishmania* species, *L. martiniquensis* and *L. orientalis*, in certain *Culicoides* species has been previously reported in endemic areas of Thailand. In Northern Thailand, *Culicoides mahasarakhamense* has been identified as the predominant vector [26, 27], while in Southern Thailand, *C. peregrinus* and *C. oxystoma* are the main putative vectors [28]. However, more than 168 *Culicoides* species from different ecotypes have been described in Southeast Asian countries, and many of them feed on mammals, which may serve as parasite reservoirs [29]. Accordingly, it can be speculated that *Leishmania* parasites may exploit more *Culicoides* species as hosts than previously reported, with spatiotemporal differences in the vector species spectrum between leishmaniasis-affected localities in different geographical regions of Thailand.

Although several experimental and field studies have implicated *Culicoides* as putative vectors of autochthonous leishmaniasis [12, 14, 26–28], evidence of their feeding behaviors on human blood has never been demonstrated, particularly in endemic areas of the country. Therefore, the incrimination of *Culicoides* as true natural vectors of human leishmaniasis according to the Killick-Kendrick criteria [30] cannot be strengthened. Several attempts were made using Sanger sequencing to analyze blood meals in engorged *Culicoides* collected near human dwellings in leishmaniasis-endemic areas, but it was found that all specimens fed only on animals with domestic and peridomestic environments, without evidence of human blood [28, 31]. We speculated that these results were likely biased because Sanger sequencing can only generate a single chromatogram, representing a single blood meal source, and is therefore not suitable for characterizing multiple blood meal sources in a single specimen. Additionally, information on the composition of *Culicoides* with seasonal abundance, prevalence of infection, and sympatric occurrence of *Leishmania* parasites in the affected communities remains limited.

In the present study, we comparatively investigated the seasonal dynamics of *Culicoides* biting midges and the infection prevalence and genetic diversity of *Leishmania* in *Culicoides* samples collected from the residence of the leishmaniasis index case in Lampang Province, an emerging focus of visceral leishmaniasis in Northern Thailand, from 2021 to 2023. In addition, we demonstrated the application of a nanopore-based metabarcoding strategy to provide high-throughput data for the unbiased identification of multiple host species. Novel insights from this study will help us better understand the role of *Culicoides* in leishmaniasis transmission and facilitate effective vector management and control to interrupt disease transmission, especially in endemic areas of northern Thailand.

## Methods

### Investigation area, biting midge collection, and morphological identification

**Fig. 1:**
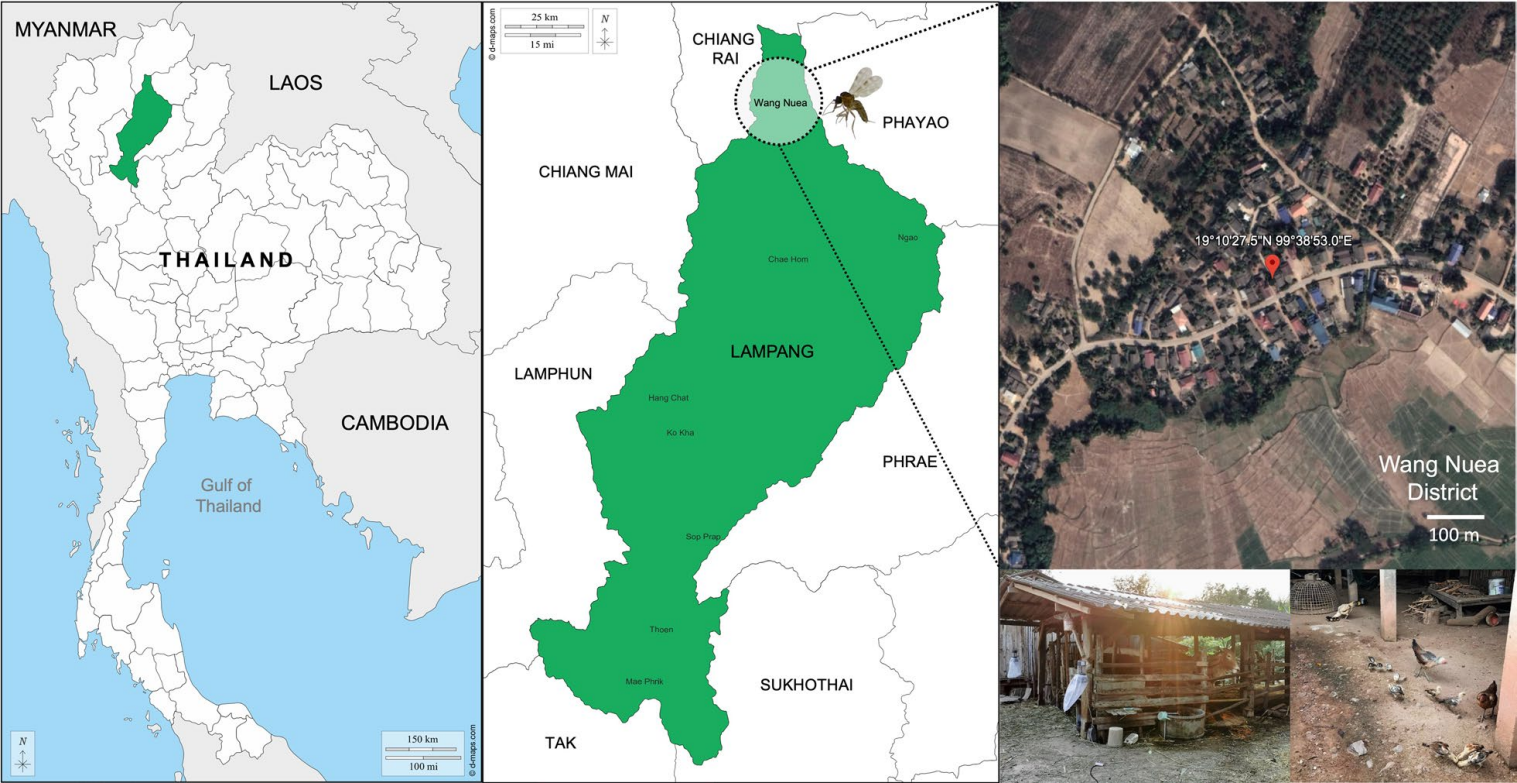
Map and geographic coordinates of *Culicoides* trapping site in Wang Nuea District, Lampang Province, Northern Thailand. The light traps were installed near the cattle pen and chicken coop within the patient’s housing area. The map of Thailand was obtained from the public domain (https://d-maps.com). The satellite image was modified from the Google Earth website (https://earth.google.com/web/search/Thailand)

*Culicoides* biting midges were collected in Wang Nuea District, Lampang Province, Northern Thailand (19° 10′ 27.5″ N, 99° 38′ 53.0″ E), from November 2021 to May 2023 (Fig. 1). The midge collection was performed within a 50-m radius around the residence of the index case who had been previously diagnosed with visceral leishmaniasis. The Centers for Disease Control and Prevention miniature UV light traps were situated 1 m above the ground in different places near the household area and neighboring cattle sheds. The traps were installed from dusk to dawn for 3 consecutive days. All insect specimens collected the next day would be immobilized by being knocked out in the freezer for 30 min. At the field site, the collection was then inspected under a stereomicroscope (EZ4 HD, Leica, Germany) to sort female *Culicoides* individuals from males and other insect species, according to distinct morphological structures, i.e., non-plumose antennae and wing venation patterns. In this study, nulliparous females, which generally predominated in the traps and had never been exposed to an infectious blood meal, were discarded from the female collection. Only non-engorged (parous and gravid) and blood-engorged ones, which had fed on hosts and entered the gonotrophic cycle, were picked up for downstream analysis. All midge specimens were then morphologically identified using the illustrated keys to *Culicoides* of Southeast Asia formerly described by Wirth and Hubert [29] and the formal description of a new species, *C. mahasarakhamense*, recorded by Pramual et al. [32]. Then, the midge specimens were frozen at – 80 °C before genomic DNA isolation.

### Genomic DNA isolation from *Culicoides* samples

To preserve the morphological structure of the samples, each *Culicoides* individual was extracted for total genomic DNA (gDNA) without sample homogenization using a non-destructive protocol previously described by Santos et al. [33]. Briefly, each sample was digested in 200 µl lysis buffer containing Proteinase K and incubated at 50 °C overnight. The lysate was then purified using the magnetic bead-based system with the GENTi^™^ Advanced Genomic DNA Extraction Kit (GeneAll^®^, Seoul, South Korea). The quality and concentration of gDNA were assessed using the NanoDrop 2000c spectrophotometer (Thermo Fisher Scientific, Waltham, MA, USA). The gDNA samples obtained were stored at – 20 °C until use. Post-extracted insect remains were stored in 70% ethanol at room temperature for further species reaffirmation.

### Initial screening of *Leishmania* DNA by *SSU rRNA*-qPCR

All extracted gDNA samples were first tested for the presence of *Leishmania* DNA using TaqMan quantitative PCR (qPCR) targeting the conserved region of the small subunit ribosomal RNA (*SSU rRNA*) gene of all *Leishmania* species, as previously described [34]. The *SSU rRNA*-qPCR reactions were performed using a forward primer (5′-CCAAAGTGTGGAGATCGAAG-3′), a reverse primer (5′-GGCCGGTAAAGGCCGAATAG-3′), and a TaqMan probe (5′-6FAM-ACCATTGTAGTCCACACTGC-3′-MGB-NFQ). Reaction components were prepared in a total volume of 20 µl containing 10 µl TaqMan^™^ Fast Advanced Master Mix (Thermo Scientific, Waltham, MA, USA), 0.5 µM each of 10 µM primers, 0.5 µl 10 µM probe, and 2 µl gDNA. All reactions were performed on the QuantStudio^™^ *5* Real-Time PCR System (Applied Biosystems, CA, USA) with the following thermal conditions: initial denaturation at 95 °C, 2 min and 45 cycles of denaturation at 95 °C, 15 s and annealing/extension at 60 °C, 15 s. A Ct value < 40 was considered positive.

### Conventional *LeishmaniaITS1*-PCR

The gDNA samples with positive qPCR results were used as templates for conventional PCR targeting all *Leishmania* species' internal transcribed spacer-1 (*ITS1*) region. *ITS1*-PCR reactions were performed using forward primer LITSR2 (5’-CTGGATCATTTTCCGATGATT-3’) and reverse primer L5.8Sinner (5’-GTTATGTGAGCCGTTATCC-3’) to generate an amplicon of approximately 272–280 bp [35]. Reaction components consisted of 4 µl gDNA, 1.5 µl each of 10 µM primers, 25 µl 2X KAPA HiFi HotStart ReadyMix (Roche, Basel, Switzerland), and nuclease-free water added to a final volume of 50 µl. Thermal conditions included initial denaturation at 95 °C, 5 min, followed by 35 cycles of denaturation at 98 °C, 30 s, annealing at 53 °C, 30 s, extension at 72 °C, 30 s, and final extension at 72 °C, 10 min. Amplification products were verified on 1.5% (*w*/*v*) agarose gel electrophoresis stained with ethidium bromide and then visualized using the Gel Doc XR + Gel Documentation System (Bio-Rad, Hercules, CA, USA).

### Parasite identification by Sanger plasmid DNA sequencing

All *Leishmania ITS1* amplicons were ligated into the pGEM® T-Easy cloning vector (Promega Corp., Madison, WI, USA) according to the manufacturer's specifications. The ligation products were then chemically transformed into competent *Escherichia coli* strain DH5α cells. Transformants were transferred onto the Luria Bertani (LB) agar plates containing ampicillin, IPTG, and X-Gal for blue/white colony selection. Five white colonies suspected of harboring the recombinant plasmids were confirmed by PCR and subsequently cultured overnight at 37 °C in LB broth medium containing ampicillin. Chimeric plasmids were extracted using the Exprep^™^ Plasmid SV DNA Purification Kit (GeneAll^®^, Seoul, Korea) and sequenced using T7 promoter primer by Macrogen, Inc. (Seoul, Korea). To identify the parasite species, the *ITS1* sequences obtained were compared with GenBank references in the nucleotide collection (nr/nt) database using BLASTn (http://blast.ncbi.nlm.nih.gov/Blast.cgi) optimized for highly similar sequences.

### Haplotype network analysis

The genetic variability of *L. martiniquensis* and *L. orientalis ITS1* sequences identified in this study and those from GenBank was investigated by haplotype network analysis based on single nucleotide variation and small insertions and deletions (indels). A minimum-spanning network was constructed in RStudio version 2024.04.2+764 [36]. Briefly, the *ITS1* sequences in FASTA format were converted into a DNA binary file required for network construction using the package “adegenet” version 2.1.10 [37]. The package “pegas” version 1.3 was then used to create the haplotype network from a list of DNA sequences in DNA binary format [38]. DNA sequences were compared, and each unique sequence was assigned to a specific haplotype using the 'haplotype' function in the pegas package. Genetic diversity statistics, including the number of haplotypes, haplotype diversity (Hd), nucleotide diversity (π), average number of nucleotide differences (k), and Tajima’s *D* statistic, were calculated using the package “pegas” version 1.3. Fu and Li’s *D* statistics were calculated using the package “PopGenome” version 2.7.5 [39]. *P*-values < 0.05 were considered statistically significant.

### PCR amplification of mammalian and avian cytochrome b regions

The gDNA extracted from engorged specimens was used as a template for PCR targeting the vertebrate cytochrome b (*Cytb*) region for mammalian and avian host identification as described elsewhere. For mammalian hosts, multiplex PCR reactions were set up in a total volume of 25 µl consisting of 3 µl gDNA template, 0.9 µl 10 µM forward primer (Human741F, Cow121F, Dog368F, Pig573F), and universal reverse primer (UNREV1025) [40], 12.5 µl 2X KAPA HiFi HotStart ReadyMix, and 5 µl nuclease-free water. Additionally, singleplex PCR reactions were performed for the avian host using L15557 and H16065 primers [41]. Thermal conditions included initial denaturation at 95 °C, 5 min; 35 cycles of denaturation at 95 °C, 1 min, annealing at 58 °C (for mammals) and 50 °C (for avian), 1 min, extension at 72 °C, 1 min, and final extension at 72 °C, 7 min. PCR products were verified by 1.5% (*w*/*v*) agarose gel electrophoresis with staining and visualization as previously described. The expected amplicon sizes were 334, 561, 680, 453, and 508 bp for human, bovine, canine, porcine, and avian, respectively.

### Vertebrate *COI*-PCR and MinION^®^ amplicon sequencing

To identify additional hosts not detected by *Cytb*-PCR, nanopore amplicon sequencing was performed on all engorged samples. The partial cytochrome c oxidase subunit I (*COI*) gene of approximately 395 bp was amplified using VertCOI_7194_F (5’-CGMATRAAYAAYATRAGCTTCTGAY-3’) and Mod_RepCOI_R (5’-TTCDGGRTGNCCRAARAATCA-3’) [42]. The amplification reactions were set up in a total volume of 25 µl consisting of 3 µl gDNA template, 0.75 µl of each 10 µM primer, and 12.5 µl 2X KAPA HiFi HotStart ReadyMix. Thermal conditions were programmed as follows: 95 °C for 3 min, 40 cycles of denaturation at 95 °C, 40 s, annealing at 48.5 °C, 30 s, extension at 72 °C, 1 min, and final extension at 72 °C for 7 min. Each amplicon sample was then purified using Agencourt AMPure XP beads (Beckman Coulter, CA, USA).

For DNA library preparation, 200 fmol purified amplicon per sample was end-repaired using the NEBNext Ultra II End Repair/dA-Tailing Module (New England Biolabs, MA, USA). End-prepped DNA samples were ligated with native barcodes provided in the Native Barcoding Kit 96 V14 (Cat. No. SQK-NBD114.96, Oxford Nanopore Technologies, Didcot, UK) using NEB Blunt/TA Ligase Master Mix (New England Biolabs). For multiplexing, barcoded samples were pooled and ligated to the sequencing adaptor using the NEBNext Quick Ligation Module (New England Biolabs). After removing the excess adaptor, 50 fmol of the final preparation was loaded into the MinION^®^ R10.4.1 flow cell for 10 h. The Dorado version 7.3.11 was used for super-accuracy base calling using the dna_r10.4.1_e8.2_400bps_sup@v.4.3.0 model, demultiplexing, and adaptor-barcode trimming. Sequenced reads with Q scores < 20 were filtered out using Chopper version 0.7.0 [43], and reads between 300–500 bp were analyzed. Reads of similar sequence and length were clustered using amplicon_sorter.py (version 2024_02_20) [44] and then error-polished with raw reads using Medaka version 1.11.3 to generate all accurate consensus sequences for each sample. For the taxonomic assignment, the obtained consensus sequences of each sample were compared to GenBank references using BLASTn optimized for highly similar sequences.

## Results

### Index case description

On 11 January 2021, a 60-year-old male living in Wang Nuea District, Lampang Province, presented with a 1-month history of fatigue and leg myalgia. Physical examination revealed markedly pale conjunctiva, consistent with anemia. Hematological investigations revealed pancytopenia with a hemoglobin level of 5.0 g/dl, hematocrit of 16%, white blood cell count of 2040 cells/mm^3^ (neutrophil, 38%; lymphocyte, 56%; monocyte, 3%; eosinophil, 2%; basophil, 1%), and platelet count of 72,000 platelets/mm^3^. His HIV serology was negative. He then received several blood transfusions without significant improvement in his symptoms, and huge splenomegaly developed. In August 2021, a bone marrow biopsy showed several macrophages full of intracellular amastigotes. *ITS1*-PCR and Sanger sequencing confirmed the final diagnosis of visceral leishmaniasis caused by *L. martiniquensis*. The DNA sequence obtained was submitted to GenBank under accession no. OR917763. The patient improved clinically following a 1-week course of intravenous amphotericin B (1 mg/kg/day) with seven doses of filgrastim (300 mcg/day) by subcutaneous injection every 2 days.

### Seasonal abundance of *Culicoides* species

**Fig. 2:**
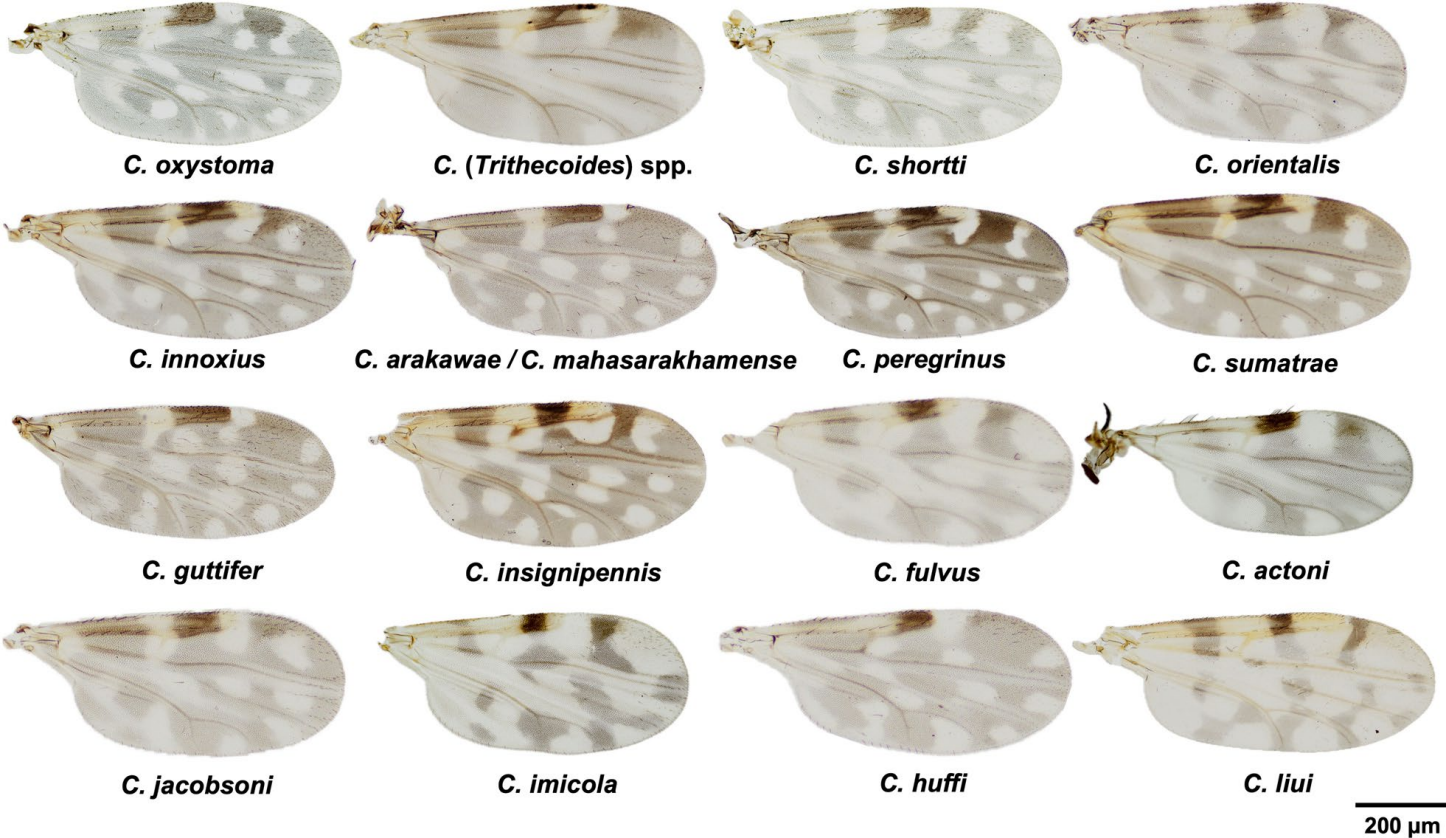
Wing morphology and pigmentation patterns of *Culicoides* species from the trap collections, shown in descending order of total relative abundance. The scale bar represents 200 µM

*Culicoides* biting midges were caught three times around the patient’s household in November 2021, March 2022, and May 2023. In total, our collection consisted of 501 non-engorged (parous and gravid) females and 51 blood-engorged females caught only in November 2021. All *Culicoides* samples were taxonomically identified as at least 17 different species belonging to five subgenera, namely *Remmia*, *Trithecoides*, *Avaritia*, *Hoffmania*, *Meijerehelea*, and two species groups, namely *Shortti* and *Calvipalpis*, mainly based on the characteristic wing pigmentation patterns (Table 1, Fig. 2). With temporal variability, *C.* (*Trithecoides*) spp. were prevalent in November 2021, whereas *C. innoxius* and *C. oxystoma* were the most abundant species in March 2022 and May 2023, respectively. Only a single specimen of *Culicoides imicola*, *C. huffi*, and *C. liui* were found sporadically during these three collection periods. The three most abundant species of the entire collection were *C. oxystoma*, *C.* (*Trithecoides*) spp., and *C. shortti*, representing 29.3%, 15.5%, and 11.9% of all *Culicoides* midges, respectively.

**Table 1:** Species diversity with temporal and overall variability in the relative abundance of *Culicoides* biting midges collected from the vicinity of the patient’s residence during three collection periods from November 2021 to May 2023

Genus (subgenus/species group)	Species name	November 2021 (winter)		March 2022 (summer)	May 2023 (rainy)	Total
		Unfed	Engorged	Unfed	Unfed	
*C.* (*Remmia*)	*oxystoma*	12	–	9	139	160 (29.3%)
*C.* (*Trithecoides*)	spp*.*	66	5	8	6	85 (15.5%)
*C.* (*Shortti* group)	*shortti*	11	18	16	20	65 (11.9%)
*C.* (*Avaritia*)	*orientalis*	46	5	–	4	55 (10.1%)
*C.* (*Hoffmania*)	*innoxius*	5	–	31	5	41 (7.5%)
*C.* (*Meijerehelea*)	*arakawae*	1	–	1	32	34 (6.2%)
*C.* (*Hoffmania*)	*peregrinus*	22	–	3	5	30 (5.5%)
*C.* (*Hoffmania*)	*sumatrae*	19	–	1	–	20 (3.7%)
*C.* (*Meijerehelea*)	*guttifer*	–	2	1	14	17 (3.1%)
*C.* (*Meijerehelea*)	*mahasarakhamense*	–	2	1	11	14 (2.6%)
*C.* (*Hoffmania*)	*insignipennis*	5	1	2	1	9 (1.6%)
*C.* (*Avaritia*)	*fulvus*	–	7	–	–	7 (1.3%)
*C.* (*Avaritia*)	*actoni*	–	5	–	–	5 (0.9%)
*C.* (*Avaritia*)	*jacobsoni*	1	1	–	–	2 (0.4%)
*C.* (*Avaritia*)	*imicola*	–	–	1	–	1 (0.2%)
*C.* (*Clavipalpis* group)	*huffi*	–	–	–	1	1 (0.2%)
*C.* (*Hoffmania*)	*liui*	–	–	1	–	1 (0.2%)
Total		188	46	75	238	547

### Infection prevalence in *Culicoides* and parasite identification

Of 501 non-engorged and 46 blood-engorged *Culicoides* samples tested, *Leishmania SSU rRNA*-qPCR was positive in 27 non-engorged and 4 engorged individuals, consisting of *C. oxystoma* (*n* = 6), *C. innoxius* (*n* = 4), *C. shortti* (*n* = 4), *C. guttifer* (*n* = 3), *C. orientalis* (*n* = 3), *C. mahasarakhamense* (*n* = 3), *C. arakawae* (*n* = 2), *C. sumatrae* (*n* = 2), *C.* (*Trithecoides*) spp. (*n* = 2), *C. actoni* (*n* = 1), and *C. fulvus* (*n* = 1), with an overall prevalence of 5.7%. *Leishmania* was temporally detected with the highest frequency in *C. orientalis*, *C. innoxius*, and *C. oxystoma* in November 2021, March 2022, and May 2023, respectively, as shown in Table 2. Conventional *ITS1*-PCR successfully amplified all qPCR-positive samples. Plasmid DNA sequencing and BLASTn analysis revealed that *L. martiniquensis* was commonly detected in 24 samples of these positive *Culicoides* species, whereas *L. orientalis* was only amplified in three samples of *C. oxystoma*. None of the samples showed co-infection with these two *Leishmania* (*Mundinia*) species. The *Leishmania ITS1* sequences obtained from non-engorged and blood-engorged samples were deposited in GenBank under accession nos. OR917764-OR917790 and PQ014661-PQ014664, respectively.

**Table 2:** Parasite identification and accession numbers of *Leishmania* sequences obtained from the patient and field-caught *Culicoides* biting midges by conventional *ITS1*-PCR and plasmid sequencing in this study

Collection date	Host species	Sample code	Parasite identification	Accession no.	BLASTn result and % identity
August 2021	*Homo sapiens* (patient)	LP-VL	*L. martiniquensis*	OR917763	*L. martiniquensis* 770,605 (KY982650), 100%
November 2021	*C. orientalis*	LP002	*L. martiniquensis*	OR917764	*L. martiniquensis* SK4-1 (MK603826), 99.2%
	*C. orientalis*	LP118	*L. martiniquensis*	OR917765	*L. martiniquensis* 770,605 (KY982650), 100%
	*C. orientalis*	LP148	*L. martiniquensis*	OR917766	*L. martiniquensis* 770,605 (KY982650), 100%
	*C.* (*Trithecoides*) sp.	LP036	*L. martiniquensis*	OR917767	*L. martiniquensis* 770,605 (KY982650), 100%
	*C.* (*Trithecoides*) sp.	LP094	*L. martiniquensis*	OR917768	*L. martiniquensis* SK4-1 (MK603826), 98.5%
	*C. sumatrae*	LP048	*L. martiniquensis*	OR917769	*L. martiniquensis* SK4-1 (MK603826), 98.1%
	*C. sumatrae*	LP129	*L. martiniquensis*	OR917770	*L. martiniquensis* SK4-1 (MK603826), 98.5%
	*C. innoxius*	LP156	*L. martiniquensis*	OR917771	*L. martiniquensis* SK4-1 (MK603826), 99.2%
	*C. fulvus*	BF02	*L. martiniquensis*	PQ014661	*L. martiniquensis* 770,605 (KY982650), 100%
	*C. actoni*	BF03	*L. martiniquensis*	PQ014662	*L. martiniquensis* 770,605 (KY982650), 100%
	*C. shortti*	BF10	*L. martiniquensis*	PQ014663	*L. martiniquensis* 770,605 (KY982650), 100%
	*C. shortti*	BF21	*L. martiniquensis*	PQ014664	*L. martiniquensis* 770,605 (KY982650), 100%
March 2022	*C. innoxius*	LP260	*L. martiniquensis*	OR917772	*L. martiniquensis* 770,605 (KY982650), 100%
	*C. innoxius*	LP272	*L. martiniquensis*	OR917773	*L. martiniquensis* TR17 (OR077858), 100%
	*C. innoxius*	LP286	*L. martiniquensis*	OR917774	*L. martiniquensis* TR17 (OR077858), 100%
	*C. shortti*	LP309	*L. martiniquensis*	OR917775	*L. martiniquensis* 770,605 (KY982650), 100%
May 2023	*C. oxystoma*	LP364	*L. martiniquensis*	OR917776	*L. martiniquensis* 770,605 (KY982650), 100%
	*C. oxystoma*	LP478	*L. martiniquensis*	OR917777	*L. martiniquensis* 770,605 (KY982650), 100%
	*C. oxystoma*	LP496	*L. martiniquensis*	OR917778	*L. martiniquensis* 770,605 (KY982650), 98.9%
	*C. oxystoma*	LP408	*L. orientalis*	OR917779	*L. orientalis* 609,106 (KY982677), 99.2%
	*C. oxystoma*	LP419	*L. orientalis*	OR917780	*L. orientalis* PCM2 (JX195640), 100%
	*C. oxystoma*	LP443	*L. orientalis*	OR917781	*L. orientalis* PCM2 (JX195640), 100%
	*C. mahasarakhamense*	LP367	*L. martiniquensis*	OR917782	*L. martiniquensis* 770,605 (KY982650), 100%
	*C. mahasarakhamense*	LP374	*L. martiniquensis*	OR917783	*L. martiniquensis* 770,605 (KY982650), 99.6%
	*C. mahasarakhamense*	LP495	*L. martiniquensis*	OR917784	*L. martiniquensis* 770,605 (KY982650), 99.2%
	*C. arakawae*	LP429	*L. martiniquensis*	OR917785	*L. martiniquensis* 770,605 (KY982650), 100%
	*C. arakawae*	LP459	*L. martiniquensis*	OR917786	*L. martiniquensis* 770,605 (KY982650), 100%
	*C. guttifer*	LP446	*L. martiniquensis*	OR917787	*L. martiniquensis* 770,605 (KY982650), 100%
	*C. guttifer*	LP508	*L. martiniquensis*	OR917788	*L. martiniquensis* 770,605 (KY982650), 99.6%
	*C. guttifer*	LP532	*L. martiniquensis*	OR917789	*L. martiniquensis* 770,605 (KY982650), 100%
	*C. shortti*	LP474	*L. martiniquensis*	OR917790	*L. martiniquensis* 770,605 (KY982650), 100%

### Haplotype diversity and neutrality statistics of detected *Leishmania*

**Fig. 3:**
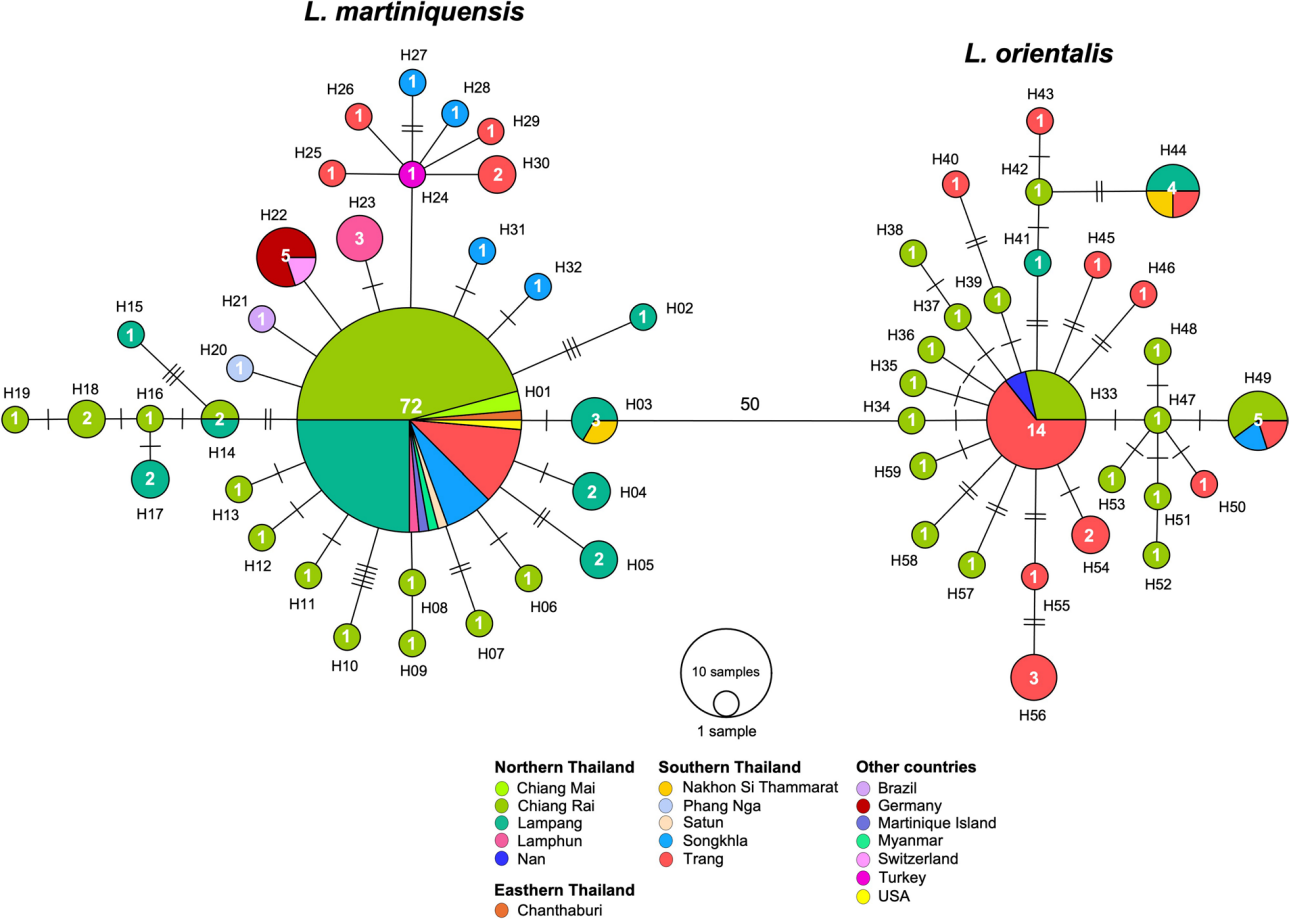
Haplotype diversity of *Leishmania* (*Mundinia*) species from different geographical origins based on *ITS1* sequences. The size of the circle indicates the number of each unique haplotype. Hatch marks between the circles represent the number of nucleotide polymorphisms between haplotypes. Blue-green circles represent unique haplotypes of *L. martiniquensis* and *L. orientalis* identified in the present study

A total of 167 *Leishmania ITS1* sequences, including 29 *L. martiniquensis* and 3 *L. orientalis* sequences amplified from the index case and *Culicoides* samples in our collection and 135 pre-exisitng sequences from the GenBank database, consisting of *L. martiniquensis* (*n* = 88) and *L. orientalis* (*n* = 47), were included in the haplotype diversity analysis. As shown in Fig. 3, the star-like network consisted of 32 and 27 unique haplotypes of *L. martiniquensis* and *L. orientalis*, respectively, circulating in *Culicoides* biting midges, animal hosts (cow, horse, and black rat), and leishmaniasis patients from Thailand and other geographical regions, as detailed in Supplementary File 1.

For *L. martiniquensis*, H01 was the most common haplotype shared by 72 sequences worldwide from several leishmaniasis-affected areas of Thailand, including Chanthaburi, Chiang Mai, Chiang Rai, Lampang (in this study), Lamphun, Nakhon Si Thammarat, Satun, Trang, and Songkhla Provinces, and other countries, namely Martinique Island, Myanmar, and the USA. The remaining descendant haplotypes were less common with varying degrees of regional area specificity. As shown in Tables 3 and 4, eight unique haplotypes with 14 polymorphic sites were identified from all *L. martiniquensis* sequences in our collection, with most sequences grouped into the dominant haplotype H01. The haplotype diversity for our collection and populations of *L. martiniquensis* in the northern provinces and the whole country was relatively high with values of 0.5556–0.6133. In contrast, nucleotide diversity for these three populations was exceptionally low with values of 0.0050–0.0063. In addition, neutrality tests showed significantly negative Tajima’s *D* values of −2.3353 and −2.5642 for the latter two populations and a significantly negative Fu and Li’s *D* value of −4.8110 for the whole country population.

**Table 3:** Haplotypes of 32 *Leishmania ITS1* sequences amplified from the index case and *Culicoides* biting midges collected in this study

*Leishmania* species	Haplotypes	No.	Accession numbers of associated *Leishmania ITS1* sequences with human or *Culicoides* hosts
*L. martiniquensis*	H01	18	OR917763 (patient), OR917765 (*C. orientalis*), OR917766 (*C. orientalis*), OR917767 (*C.* (*Trithecoides*) sp.), OR917772 (*C. innoxius*), OR917775 (*C. shortti*), OR917776 (*C. oxystoma*), OR917777 (*C. oxystoma*), OR917782 (*C. mahasarakhamense*), OR917785 (*C. arakawae*), OR917786 (*C. arakawae*), OR917787 (*C. guttifer*), OR917789 (*C. guttifer*), OR917790 (*C. shortti*) PQ014661 (*C. fulvus*), PQ014662 (*C. actoni*) PQ014663 (*C. shortti*), PQ014664 (*C. shortti*)
	H02	1	OR917778 (*C. oxystoma*)
	H03	2	OR917773 (*C. innoxius*), OR917774 (*C. innoxius*)
	H04	2	OR917783 (*C. mahasarakhamense*), OR917788 (*C. guttifer*)
	H05	2	OR917764 (*C. orientalis*), OR917771 (*C. innoxius*)
	H14	1	OR917784 (*C. mahasarakhamense*)
	H15	1	OR917769 (*C. sumatrae*)
	H17	2	OR917768 (*C.* (*Trithecoides*) sp.), OR917770 (*C. sumatrae*)
*L. orientalis*	H41	1	OR917779 (*C. oxystoma*)
	H44	2	OR917780 (*C. oxystoma*), OR917781 (*C. oxystoma*)

**Table 4:** Genetic diversity and neutrality statistics of *Leishmania martiniquensis* and *L. orientalis* populations identified in *Culicoides* vectors and humans in different geographic localities of Thailand based on *ITS1* region

	Population	Total sample size (n)	No. haplotypes (H)	No. polymorphic sites (S)	Average no. of nucleotide difference (k)	Haplotype diversity (Hd) ± SD	Nucleotide diversity (π) ± SD	Tajima's *D*	Fu and Li's *D*
*L. martiniquensis*	Lampang Province - *Culicoides* (28) and the index case (1)	29	8	14	1.6256	0.6133 ± 0.1025	0.0063 ± 0.0042	−1.8282^NS^	−1.3159^NS^
	Northern Region - *Culicoides* (76) and humans (5)	81	20	27	1.3222	0.5556 ± 0.0680	0.0051 ± 0.0035	−2.3353*	−3.5626**
	Thailand - *Culicoides* (82), black rat (1), and humans (24)	107	29	39	1.2756	0.5842 ± 0.0585	0.0050 ± 0.0034	−2.5642*	−4.8110**
*L. orientalis*	Lampang Province - Culicoides (3)	3	2	5	2.0000	0.6667 ± 0.2722	0.0080 ± 0.0073	NA	NA
	Northern Thailand - *Culicoides* (25) and humans (1)	26	19	25	2.0800	0.9569 ± 0.0259	0.0082 ± 0.0051	−2.4975*	−1.8429^NS^
	Thailand - *Culicoides* (26) and humans (24)	50	27	31	2.1396	0.9094 ± 0.0318	0.0086 ± 0.0052	−2.3052*	−2.9282**

^*^p-value < 0.05; **p-value < 0.01; *NS* not significant; *NA* not analyzed

For *L. orientalis*, the H33 haplotype was the most dominant with 14 sequences from Chiang Rai and Nan in Northern Thailand and Trang Province in Southern Thailand. Two haplotypes (H41 and H44) with five polymorphic sites were identified in our collection. Two haplotypes, H44 and H49, could be found in different geographical locations. H44 was shared by four individuals from Lampang, Nakhon Si Thammarat, and Trang Provinces, while H49 was shared by five individuals from Chiang Rai, Trang, and Songkhla Provinces. This suggests possible gene flow across these sites. The haplotype diversity value for our collection was 0.6667, while those of *L. orientalis* populations in the northern region and the whole country were remarkably higher with values of 0.9569 and 0.9094, respectively. The lower degree of genetic differentiation of *L. orientalis* in our collection was probably due to the considerably small effective population size. As with *L. martiniquensis*, nucleotide diversity values for these groups ranged from 0.0080 to 0.0086. Neutrality tests were also significantly negative, with Tajima’s *D* values of − 2.4975 and − 2.3052 for the northern region and the whole country populations, respectively, and Fu and Li’s D value of − 2.9282 for the whole country population.

### Blood meal source identification by host-specific *Cytb*-PCRs

A total of 46 blood-engorged females of nine *Culicoides* species were collected in November 2021 as detailed in Table 1. The *Cytb*-PCR reactions targeting mammalian and avian DNA were successfully performed on all engorged samples. Of these, four kinds of vertebrate hosts were identified with the following detection frequencies: cow (*n* = 41), human (*n* = 7), bird (*n* = 4), and pig (*n* = 1). Forty-one samples with cows as hosts consisted of *Culicoides shortti* (*n* = 18), *C. fulvus* (*n* = 7), *C. orientalis* (*n* = 4), *C. actoni* (*n* = 5), *C.* (*Trithecoides*) spp. (*n* = 5), *C. jacobsoni* (*n* = 1), and *C. insignipennis* (*n* = 1). Of these 41 samples, six samples of four species, namely *C. shortti* (*n* = 3), *C. fulvus* (*n* = 1), *C. orientalis* (*n* = 1), and *C. actoni* (*n* = 1), were found to feed on both cows and humans. Pig blood was detected in only one sample of *C. orientalis*, while none of the samples tested positive for dogs. Four samples of two *Culicoides* species, *C. guttifer* (*n* = 2) and *C. mahasarakhamense* (*n* = 2), were recorded as feeding on birds, and one of these two *C. guttifer* samples also fed on humans.

### MinION^®^ amplicon sequencing

**Fig. 4:**
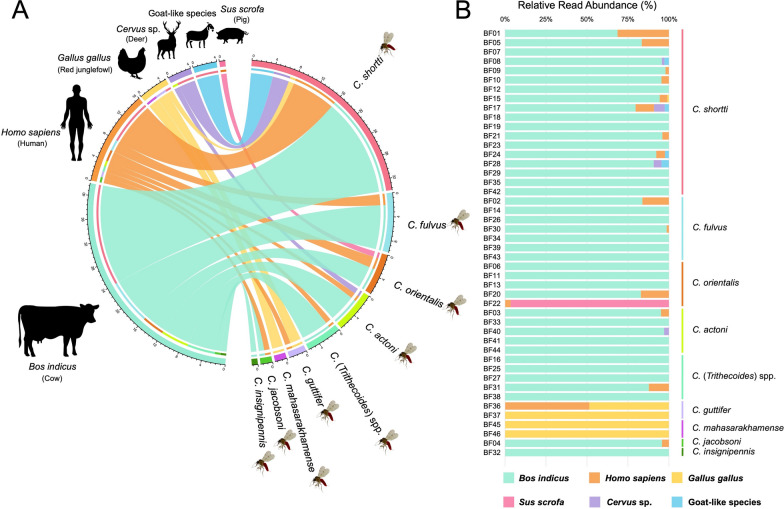
(**A**) Circos plot illustrating the association between blood meal origins and each *Culicoides* species. (**B**) Bar chart showing the relative abundance of sequenced reads in 46 blood-engorged *Culicoides* samples analyzed by MinION^®^ amplicon sequencing in this study

A metabarcoding approach using nanopore-based *COI* amplicon sequencing was also used to generate high-throughput data, greatly facilitating the identification of multiple host species. *COI*-PCR was also successfully performed on all the engorged samples in which host-specific *Cytb*-PCRs were obtained. After filtering out low-quality reads, 739,245 high-quality reads with Q scores ≥ 20 were obtained from all individuals in the engorged collection. The resulting high-quality reads from each sample were clustered and error-polished to generate highly accurate consensus sequences. In total, 71 consensus sequences were generated and aligned against GenBank references using BLASTn for the host taxonomic assignment of each sample as detailed in Table 5. As shown in Fig. 4, six vertebrate host species were identified with the following detection frequencies: *Bos indicus* (cow, *n* = 41), *Homo sapiens* (human, *n* = 16), *Gallus gallus* (chicken, *n* = 5), *Cervus* sp. (deer, *n* = 4), and *Sus scrofa* (pig, *n* = 1), with high similarity percentages ranging from 94.7–99.3%, and unknown species related to *Rupicapra* sp. (goat-like species, *n* = 4) with similarity percentages of 89.9–90.5%.

**Table 5:** The information on sources of blood meals in 46 blood-engorged *Culicoides* samples identified by host-specific *Cytb*-PCR and MinION® amplicon sequencing

**Species**	**Sample ID**	***Leishmania*** **status**	**Host-specific *Cytb*-PCR**					**Vertebrate *COI*-PCR and MinION® sequencing**				
			**Cow**	**Human**	**Pig**	**Dog**	**Bird**	**BLASTn result**	**No. assigned** **reads**	**Relative abundance (%)**	**E value**	**Percent** **Identity**
*C. shortti*	BF07	Negative	+	–	–	–	–	*Bos indicus*	24,347	100	0	98.9
*C. shortti*	BF12	Negative	+	–	–	–	–	*Bos indicus*	14,260	100	0	98.9
*C. shortti*	BF18	Negative	+	–	–	–	–	*Bos indicus*	36,482	100	0	98.6
*C. shortti*	BF19	Negative	+	–	–	–	–	*Bos indicus*	24,036	100	0	98.6
*C. shortti*	BF23	Negative	+	–	–	–	–	*Bos indicus*	17,571	100	0	98.6
*C. shortti*	BF29	Negative	+	–	–	–	–	*Bos indicus*	24,789	100	0	98.6
*C. shortti*	BF35	Negative	+	–	–	–	–	*Bos indicus*	35,262	100	0	98.4
*C. shortti*	BF42	Negative	+	–	–	–	–	*Bos indicus*	25,506	100	0	98.9
*C. fulvus*	BF14	Negative	+	–	–	–	-	*Bos indicus*	17,190	100	0	98.6
*C. fulvus*	BF26	Negative	+	–	–	–	–	*Bos indicus*	41,004	100	0	98.9
*C. fulvus*	BF34	Negative	+	–	–	–	–	*Bos indicus*	128	100	0	99.1
*C. fulvus*	BF39	Negative	+	–	–	–	–	*Bos indicus*	390	100	0	98.6
*C. fulvus*	BF43	Negative	+	–	–	–	–	*Bos indicus*	774	100	0	98.8
*C.* (*Trithecoides*) sp.	BF16	Negative	+	–	–	–	–	*Bos indicus*	18,375	100	0	98.4
*C.* (*Trithecoides*) sp.	BF25	Negative	+	–	–	–	–	*Bos indicus*	17,822	100	0	98.4
*C.* (*Trithecoides*) sp.	BF27	Negative	+	–	–	–	–	*Bos indicus*	33,602	100	0	98.8
*C.* (*Trithecoides*) sp.	BF38	Negative	+	–	–	–	–	*Bos indicus*	7,642	100	0	99.1
*C. orientalis*	BF06	Negative	+	–	–	–	–	*Bos indicus*	20,214	100	0	98.6
*C. orientalis*	BF11	Negative	+	–	–	–	–	*Bos indicus*	7,268	100	0	98.6
*C. orientalis*	BF13	Negative	+	–	–	–	–	*Bos indicus*	21,969	100	0	98.9
*C. actoni*	BF33	Negative	+	–	–	–	–	*Bos indicus*	1,247	100	0	98.6
*C. actoni*	BF41	Negative	+	–	–	–	–	*Bos indicus*	10,885	100	0	99.3
*C. actoni*	BF44	Negative	+	–	–	–	–	*Bos indicus*	2,111	100	0	99.1
*C. mahasarakhamense*	BF45	Negative	–	–	–	–	+	*Gallus gallus*	8,509	100	0	98.9
*C. mahasarakhamense*	BF46	Negative	–	–	–	–	+	*Gallus gallus*	2,623	100	0	98.9
*C. guttifer*	BF37	Negative	–	–	–	–	+	*Gallus gallus*	8,693	100	0	99.1
*C. insignipennis*	BF32	Negative	+	–	–	–	–	*Bos indicus*	18,772	100	0	98.6
*C. shortti*	BF01	Negative	+	+	–	–	–	*Bos indicus*	814	68.7	0	99.1
								*Homo sapiens*	371	31.3	0	98.6
*C. shortti*	BF05	Negative	+	+	–	–	–	*Bos indicus*	10,354	83.4	0	98.6
								*Homo sapiens*	2,055	16.6	0	98.6
*C. shortti*	BF09	Negative	+	-	–	–	–	*Bos indicus*	14,663	97.9	0	98.6
								*Homo sapiens*	317	2.1	0	98.4
*C. shortti*	BF10	*L. martiniquensis*	+	+	–	–	–	*Bos indicus*	17,182	95.4	0	98.6
								*Homo sapiens*	823	4.6	0	98.6
*C. shortti*	BF21	*L. martiniquensis*	+	–	–	–	–	*Bos indicus*	7,293	96	0	98.4
								*Homo sapiens*	303	4	0	98.6
*C. fulvus*	BF02	*L. martiniquensis*	+	+	–	–	–	*Bos indicus*	23,590	83.8	0	98.4
								*Homo sapiens*	4,565	16.2	0	98.6
*C. fulvus*	BF30	Negative	+	–	–	–	–	*Bos indicus*	21,232	98.6	0	98.6
								*Homo sapiens*	298	1.4	0	98.6
*C. orientalis*	BF20	Negative	+	+	–	–	–	*Bos indicus*	16,632	82.9	0	98.6
								*Homo sapiens*	3,422	17.1	0	98.6
*C. orientalis*	BF22	Negative	–	–	+	–	–	*Sus scrofa*	9,465	96.6	0	98.8
								*Homo sapiens*	336	3.4	0	98.4
*C. actoni*	BF03	*L. martiniquensis*	+	+	–	–	–	*Bos indicus*	3796	95.2	0	99.3
								*Homo sapiens*	193	4.8	0	98.6
*C. actoni*	BF40	Negative	+	–	–	–	–	*Bos indicus*	21,723	97.1	0	98.4
								*Cervus* sp.	648	2.9	7e-177	94.7
*C.* (*Trithecoides*) sp.	BF31	Negative	+	–	–	–	–	*Bos indicus*	3927	87.8	0	98.9
								*Homo sapiens*	547	12.2	0	98.4
*C. guttifer*	BF36	Negative	–	+	–	–	+	*Homo sapiens*	11,942	51.5	0	98.6
								*Gallus gallus*	11,263	48.5	0	98.9
*C. jacobsoni*	BF04	Negative	+	–	–	–	–	*Bos indicus*	19,366	95.8	0	98.6
								*Homo sapiens*	856	4.2	0	98.6
*C. shortti*	BF08	Negative	+	–	–	–	-	*Bos indicus*	11,453	95.7	0	98.4
								*Rupicapra* sp.	328	2.7	5e-93	89.9
								*Cervus* sp.	192	1.6	2e-177	94.7
*C. shortti*	BF15	Negative	+	–	–	–	–	*Bos indicus*	9800	94.4	0	98.6
								*Homo sapiens*	479	4.6	0	98.6
								*Gallus gallus*	106	1	0	99.1
*C. shortti*	BF24	Negative	+	–	–	–	–	*Bos indicus*	17,122	92.3	0	98.6
								*Homo sapiens*	993	5.4	0	98.6
								*Rupicapra* sp.	442	2.4	4e-108	89.9
*C. shortti*	BF28	Negative	+	–	–	–	–	*Bos indicus*	26,170	90.8	0	98.4
								*Cervus* sp.	1362	4.7	2e-177	94.7
								*Rupicapra* sp.	1299	4.5	1e-124	90.5
*C. shortti*	BF17	Negative	+	–	–	–	–	*Bos indicus*	15,967	79.6	0	98.6
								*Homo sapiens*	2250	11.2	0	98.6
								*Cervus* sp.	1327	6.6	7e-177	94.7
								*Rupicapra* sp.	508	2.5	1e-124	90.5

### Performance comparison between host-specific *Cytb*-PCR and MinION^®^ amplicon sequencing

Although there were differences in the frequency of hosts detected between host-specific *Cytb*-PCR and nanopore amplicon sequencing, the most common host was cow, identified in 41 of 46 samples (89.1%) by both techniques. The second most common host was human, 15.2% by host-specific *Cytb*-PCR and 34.8% by amplicon deep sequencing. The superiority of amplicon deep sequencing in generating the high-throughput sequenced read dataset allowed the identification of additional hosts not detected by host-specific *Cytb*-PCR and showed evidence of multiple blood meal origins within certain individuals of the engorged collection as shown in Table 5. *Cytb*-PCR showed evidence of two host species in only seven samples (15.2%). In contrast, amplicon deep sequencing identified 19 samples (41.3%) with multiple hosts, including 14 with two hosts, four with three hosts, and one with four hosts. Peridomestic species (cow, chicken, and pig), humans, and wildlife (deer and unknown species related to *Rupicapra* sp.) were identified as host species in *Culicoides* samples with evidence of multiple blood meals. Of the 11 samples with cow and human as a blood source, four samples, consisting of two *C. shortti*, one *C. actoni*, and one *C. fulvus*, were found positive for *L. martiniquensis*.

## Discussion

The increasing prevalence of autochthonous human leishmaniasis, particularly in transmission areas of Thailand, has highlighted the importance of identifying vector and animal reservoir species and developing vector management strategies for effective transmission interruption. Recent experimental evidence has demonstrated successful promastigote development of several *Leishmania* (*Mundinia*) strains in laboratory-colonized *C. sonorensis*, suggesting that *Culicoides* biting midges are the imperative vectors of *Leishmania* (*Mundinia*) species rather than phlebotomine sand flies, which are traditionally known to be vectors of leishmaniasis [12–14]. However, biological information on the species diversity, population dynamics, infection prevalence, and host feeding patterns of *Culicoides* vectors remains poorly understood.

Temporal variability in species composition and abundance was observed in this study, suggesting that seasonality may influence the availability of favorable habitats for larvae of each *Culicoides* species. All *Culicoides* species were caught with the lowest abundance in summer with *C. innoxius* as the dominant species, whereas *C. oxystoma* and *C.* (*Trithecoides*) spp. were found to be the dominant species during the rainy season and winter, respectively. This could be because the summer could have contributed to the dryness of the soil habitats, strongly affecting immature *Culicoides* which require a moist environment and optimal environmental factors for their oviposition, larval development, and adult emergence [45–47], thus reducing the number of *Culicoides* populations during this period. In addition to seasonality, host availability and wind speed may also have influenced the species richness and abundance of *Culicoides* during these three collection periods [48–51].

This study demonstrated the concordance between *SSU rRNA*-qPCR and conventional *ITS1*-PCR results, showing *Leishmania* prevalence in at least 11 midge species, namely *C. oxystoma*, *C. guttifer*, *C. orientalis*, *C. mahasarakhamense*, *C* (*Trithecoides*) spp., *C. innoxius*, *C. shortti*, *C. arakawae*, *C. sumatrae, C. actoni*, and *C. fulvus*, with an overall prevalence of infection of 5.7%. *Culicoides mahasarakhamense* was first recorded as a vector of *L. martiniquensis* in Lamphun Province, Northern Thailand, in 2021 [26]. More recently, *C. mahasarakhamense*, *C. guttifer*, *C.* (*Trithecoides*) spp., *C. jacobsoni*, *C. oxystoma*, and *C. orientalis* have been implicated in the transmission of leishmaniasis in Chiang Rai Province, the most endemic area of leishmaniasis in Northern Thailand [27]. Therefore, the detection of *Leishmania* in the latter six species in our study represents the new records in Northern Thailand. Of note, *L. martiniquensis* was ubiquitously detected in all positive species over three seasonal periods with a high prevalence in the rainy season, followed by winter, whereas *L. orientalis* was only positive in three samples of *C. oxystoma* in the rainy season. Importantly, our finding indicates the sympatric occurrence of these two *Leishmania* (*Mundinia*) species circulating in the major livestock-associated biting midges in the emerging focus of leishmaniasis in Northern Thailand, suggesting that they may share *Culicoides* vector species for transmission to humans and animal reservoirs.

It could also be observed that *Leishmania* was detected with the highest frequency in the predominant species at a given collection time. It can be assumed that *Leishmania* parasites are transmitted to mammalian hosts through the bites of infected vectors; therefore, the maintenance of parasite transmission in nature appears to be closely associated with vector abundance [28, 47, 52, 53]. On the other hand, dominant vector species are more likely to increase the opportunities for pathogen transmission than less abundant species. Our findings are also consistent with the previous literature, which reported that the most abundant *Culicoides* species identified on cattle farms in Southern Ireland were associated with the transmission of arboviral diseases, including bluetongue and Schmallenberg viruses [54].

The intraspecific genetic diversity of *L. martiniquensis* and *L. orientalis* circulating in *Culicoides* biting midges, humans, and animal reservoirs in Thailand and other countries was revealed by the haplotype network of the *ITS1* region in this study. Intriguingly, the haplotype network of each parasite species exhibited a star-like distribution with a dominant central haplotype and its multiple descendant lineages with few base differences, suggesting demographic expansion following the genetic bottleneck effect [55]. Negative neutrality statistics also signified that populations of these two parasites, particularly at regional and country levels, were likely to have undergone recent selective sweeps or population expansions [56, 57]. Essentially, the genetic divergence of the dominant core haplotype into novel descendent haplotypes appears to represent an evolutionary process by which the parasites have successfully adapted to a wide range of insect and mammalian host species in different geographical locations [58, 59].

For *L. martiniquensis*, the dominant haplotype H01 was the most ancestral and widespread globally, being shared by several individuals from different geographical origins. The existence of allopatric *L. martiniquensis* isolates with the H01 haplotype in different geographical locations strongly supports the hypothesis of a supercontinent origin, suggesting that the species divergence of the subgenus *Mundinia* occurred before the break-up of Gondwana [60, 61]. More importantly, haplotype H01 contained the sequences amplified from *Culicoides* samples in our collection and all leishmaniasis patients in Northern Thailand recorded to date, including the index case in this study. This genetic similarity confirms that natural *Culicoides* individuals are infected with the same *Leishmania* parasites circulating in humans in the same transmission area. Contrarily, *L. orientalis* was identified exclusively in Thailand [5], with populations of this species in the Northern region and throughout the country exhibiting significantly higher haplotype diversity than those of *L. martiniquensis*. Our results are consistent with previous literature showing considerable genetic differentiation of *L. orientalis* populations circulating in *Culicoides* and asymptomatic seropositive individuals in Chiang Rai Province [27, 62].

As previously published, the characterization of blood meal sources of *Culicoides* collected from different geographical locations in Thailand was based on conventional PCR targeting the mitochondrial *Cytb* and *COI* genes [28, 31, 48, 63] or the nuclear prepronociceptin (*PNOC*) gene [64], followed by Sanger sequencing. To our knowledge, domestic and peridomestic animals, including cattle, dogs, goats, and chickens, were identified as hosts of *Culicoides* species collected from areas of leishmaniasis endemicity [28, 31]. Notably, only three species, *Culicoides brevitarsis*, *C. imicola*, and *C. oxystoma*, collected from non-endemic areas, were recorded as opportunistic human blood-feeders [48, 64]. Thus, evidence of human blood feeding in leishmaniasis-endemic areas has never been demonstrated. As mentioned in the Background section, Sanger-based blood meal identification appears to be more prone to bias because this traditional sequencing method could only generate a single sequence for an individual sample. Multiplex *Cytb*-PCR is another good option for the identification of common vertebrate host species due to its high sensitivity and accuracy. However, this assay cannot identify wildlife species because of the limited number of primer pairs used. We therefore circumvent this by using a nanopore-based metabarcoding approach, which can differentiate long-read amplicon populations in samples of multiple origins to identify host species with higher taxonomic resolution without bias.

The vertebrate *COI* metabarcoding dataset revealed that all nine *Culicoides* species in our study are predominantly maintained by cows, followed by humans, chickens, deer, goat-like species, and pigs. Seven *Culicoides* species, namely *C. shortti*, *C. fulvus*, *C. orientalis*, *C. actoni*, *C.* (*Trithecoides*) sp., *C. guttifer*, and *C. jacobsoni*, represent the new records of feeding on human blood with percentages of human sequence reads ranging from 1.4 to 31.3% and exceptionally 51.5% in one sample of *C. guttifer*. However, most human feeding samples have a low percentage of human sequence reads, reflecting that human feeding is less frequent or opportunistic. This may be because most *Culicoides* species tend to be crepuscular in activity [29, 65]. They forage most actively at dawn and dusk and also bite at night when humans are in settlements and unlikely to be exposed to their bites. Our metabarcoding data are consistent with previous studies [31, 48, 63], showing that two *C. guttifer* and two *C. mahasarakhamense* samples in our engorged collection are ornithophilic and feed mainly on chickens.

Previously, *L. martiniquensis* has been reported to cause cutaneous leishmaniasis in cattle in Switzerland [66] and horses in Germany [67] and the USA [68]. In addition to cattle and horses, *L. martiniquensis* was molecularly detected in the visceral tissues of black rats captured in the endemic area of Songkhla Province, Southern Thailand [69], and in the buffy coat of a black rat from the patient’s residence in Chiang Rai Province [11]. Furthermore, *Leishmania* antibodies were also found in water buffaloes, dogs, and black rats from Chiang Rai Province [11]. These molecular and serological findings implicated domestic and peridomestic animals as reservoirs of autochthonous *Leishmania* parasites. In this study, blood-engorged *Culicoides* samples were also analyzed for *Leishmania* to shed light on the role of vertebrate hosts in the transmission cycle. Interestingly, four samples in our engorged collection, consisting of two *C. shortti*, one *C. fulvus*, one *C. actoni*, contained cow and human blood and all tested positive for *L. martiniquensis*. This strongly suggests that cattle may serve as an animal reservoir for *L. martiniquensis*, posing the risk of zoonotic transmission in this transmission area.

Our nanopore-based blood meal analysis provided promising evidence that *Culicoides* in leishmaniasis-endemic areas feed on humans and animal reservoirs, supporting the first criterion of Killick-Kendrick [30]. In addition, *Leishmania* parasites identified in *Culicoides* in this study were found to be genetically identical to those from patients in the same geographical area. This finding confirms Killick-Kendrick’s third criterion, which states that parasites from vectors and vertebrate hosts are indistinguishable [30]. Accordingly, our molecular evidence supports the role of positive *Culicoides* species as putative vectors of leishmaniasis. However, it remains insufficient to incriminate *Culicoides* as true natural vectors of *Leishmania* (*Mundinia*) parasites in Thailand, as parasite DNA may be detectable after host feeding, but parasite development and transmission may not occur [24]. To prove the major involvement of Thai *Culicoides* species in leishmaniasis transmission cycles, the full development of *Leishmania* (*Mundinia*) parasites in incriminated *Culicoides* species, at least by dissection of field-caught specimens and more ideally in controlled laboratory experiments, and the capability to transmit to the vertebrate hosts need to be demonstrated, which will also fulfill the second and fourth criteria of Killick-Kendrick [12–14, 24, 25, 30]. Therefore, colonization, experimental infection, and transmission of suspected vector species will be the next steps to confirm *Culicoides* as proven vectors responsible for the autochthonous transmission of *Leishmania* (*Mundinia*) parasites in Thailand.

Finally, our results revealed the seasonal population dynamics and host preference of *Culicoides* midge populations, as well as the infection prevalence, and haplotype diversity of two *Leishmania* (*Mundinia*) species sympatrically circulating in different *Culicoides* vector species in the newly emerging focus of leishmaniasis in Northern Thailand. The novel data from this study are an important step towards a more complete understanding of *Culicoides* midges and their medical importance in the transmission of leishmaniasis in areas of endemicity in Thailand as well as effective prevention and control of this neglected parasitic disease.

## Conclusions

In this research, we investigated the species diversity, seasonal dynamics of *Culicoides* biting midges, and infection prevalence and genetic diversity of *L. martiniquensis* and *L. orientalis* circulating in different *Culicoides* species in the emerging focus of visceral leishmaniasis in Lampang Province, Northern Thailand. Our molecular findings revealed that *L. martiniquensis* was ubiquitously identified in at least 11 *Culicoides* species, whereas *L. orientalis* was only detected in *C. oxystoma*. Haplotype diversity analysis indicated recent population divergence of these two parasite species. The *L. martiniquensis* isolate identified in our index case was genetically identical to those circulating in *Culicoides* in the same area. Evidence of human blood feeding has been demonstrated, supporting the putative role of *Culicoides* in the autochthonous transmission of human leishmaniasis in Thailand. Blood meal analysis also suggested that peridomestic and wild animals, including cattle, deer, goat-like species, and pigs, may serve as parasite reservoirs with a risk of zoonotic transmission. Confirmation of metacyclic development by dissection of field-caught specimens, as well as colonization of Thai *Culicoides* species and experimental infection with *Leishmania* (*Mundinia*) parasites under laboratory conditions, will be performed in the next study. Essentially, the novel information from this study will facilitate a better understanding of the complexity of leishmaniasis transmission in endemic areas of Thailand and be a step towards developing vector control strategies to mitigate the endemicity of this neglected tropical disease effectively.

## Supplementary Information

Additional file 1: Table S1. The information on 167 *ITS1* sequences of *Leishmania* *martiniquensis *and *L. orientalis* isolates in humans, *Culicoides *biting midges, and animal hosts from Thailand and other geographical regions.

## Data Availability

All data supporting the findings of this study are available within the paper and its Supplementary Information. The *Leishmania* *ITS1* sequences obtained in this study have been uploaded into GenBank with assigned accession numbers OR917763-OR917790 and PQ014661-PQ014664.

## Abbreviations

UV: Ultraviolet
RNA: Ribonucleic acid
DNA: Deoxyribonucleic acid
qPCR: Quantitative polymerase chain reaction
PCR: Polymerase chain reaction
Ct: Cycle threshold
IPTG: Isopropyl-beta-D-1-thiogalactopyranoside
X-gal: 5-Bromo-4-chloro-3-indolyl-beta-D-galactoside
HIV: Human immunodeficiency virus
