# Supplementary material for: Seasonal dynamics, Leishmania diversity, and nanopore-based metabarcoding of blood meal origins in Culicoides spp. in the newly emerging focus of leishmaniasis in Northern Thailand
Source: Parasit Vectors. 2024 Sep 19;17:400. doi: 10.1186/s13071-024-06487-z (PMC11414243; doi:10.1186/s13071-024-06487-z)
Supplement: Supplementary file 1 — Additional file 1: Table S1. The information on 167 ITS1 sequences of Leishmania martiniquensis and L. orientalis isolates in humans, Culicoides biting midges, and animal hosts from Thailand and other geographical regions. [file 13071_2024_6487_MOESM1_ESM.docx]

**Table S1**. The information on 167 *ITS1* sequences of *L.* *martiniquensis* and *L. orientalis* isolates in humans, *Culicoides* biting midges, and animal hosts from Thailand and other geographical regions.

| *Leishmania* species | Haplotype | Country/Provinces | Host | Accession numbers | References |
| --- | --- | --- | --- | --- | --- |
| *L. martiniquensis* | H01 | Chiang Rai | *Culicoides* spp. | PP586221 (32) | Ampol et al., 2024 |
|  |  |  | Human | OM688240 | Srivarasat et al., 2022 |
|  |  | Lampang | *Culicoides* spp. | OR917765 OR917766 OR917767  OR917772 OR917775 OR917776 OR917777 OR917782 OR917785 OR917786  OR917787 OR917789 OR917790  PQ014661  PQ014662  PQ014663  PQ014664 | This study |
|  |  |  | Human | OR917763 | This study |
|  |  | Trang | Human | KY982639  KY982640  KY982641  KY982642  KY982645  KY982646  KY982650 | Manomat et al., 2017 |
|  |  |  |  | JQ001752 | Chusri et al., 2012 |
|  |  | Songkhla | Human | OP698049  OP698050 | Songumpai et al., 2022 |
|  |  |  | *Culicoides peregrinus* | OP698052 | Songumpai et al., 2022 |
|  |  |  | Human | JQ001751 | Chusri et al., 2012 |
|  |  |  | Black rat | JQ866906 | Chusri et al., 2012 |
|  |  | Chanthaburi | Human | GQ226034 | Suankratay et al., 2010 |
|  |  | Chiang Mai | Human | KJ210834  KJ210836 | Chiewchanvit et al., 2015 |
|  |  | Satun | Human | JX195637 | Leelayoova et al., 2013 |
|  |  | Lamphun | Human | JX898938 | Pothirat et al., 2014 |
|  |  | USA | Horse | JQ617283 | Reuss et al., 2012 |
|  |  | Myanmar | Human | KF211417 | Noppakun et al., 2014 |
|  |  | Martinique Island | Human | KM677931 | Pothirat et al., 2014 |
|  | H02 | Lampang | *Culicoides oxystoma* | OR917778 | This study |
|  | H03 | Lampang | *Culicoides innoxius* | OR917773  OR917774 | This study |
|  |  | Nakhon Si Thammarat | *Culicoides peregrinus* | OR077858 | Kaewmee et al. 2023 |
|  | H04 | Lampang | *Culicoides* spp. | OR917783  OR917788 | This study |

**Table S1**. The information on 167 *ITS1* sequences of *L.* *martiniquensis* and *L. orientalis* isolates in humans, *Culicoides* biting midges, and animal hosts from Thailand and other geographical regions. (Continued)

| *Leishmania* species | Haplotype | Country/Provinces | Host | Accession numbers | References |
| --- | --- | --- | --- | --- | --- |
| *L. martiniquensis* | H05 | Lampang | *Culicoides* spp. | OR917764  OR917771 | This study |
|  | H06 | Chiang Rai | *Culicoides mahasarakhamense* | PP586227 | Ampol et al., 2024 |
|  | H07 | Chiang Rai | *Culicoides guttifer* | PP586233 | Ampol et al., 2024 |
|  | H08 | Chiang Rai | *Culicoides jacobsoni* | PP586225 | Ampol et al., 2024 |
|  | H09 | Chiang Rai | *Culicoides* (*Trithecoides*) sp. | PP586226 | Ampol et al., 2024 |
|  | H10 | Chiang Rai | *Culicoides mahasarakhamense* | PP586228 | Ampol et al., 2024 |
|  | H11 | Chiang Rai | *Culicoides* (*Trithecoides*) sp. | PP586224 | Ampol et al., 2024 |
|  | H12 | Chiang Rai | *Culicoides oxystoma* | PP586222 | Ampol et al., 2024 |
|  | H13 | Chiang Rai | *Culicoides* (*Trithecoides*) sp. | PP586223 | Ampol et al., 2024 |
|  | H14 | Chiang Rai | *Culicoides* (*Trithecoides*) *sp.* | PP586230 | Ampol et al., 2024 |
|  |  | Lampang | *Culicoides mahasarakhamense* | OR917784 | This study |
|  | H15 | Lampang | *Culicoides sumatrae* | OR917769 | This study |
|  | H16 | Chiang Rai | *Culicoides* (*Trithecoides*) sp. | PP586229 | Ampol et al., 2024 |
|  | H17 | Lampang | *Culicoides* spp. | OR917768  OR917770 | This study |
|  | H18 | Chiang Rai | *Culicoides* spp. | PP586231 (2) | Ampol et al., 2024 |
|  | H19 | Chiang Rai | *Culicoides guttifer* | PP586232 | Ampol et al., 2024 |
|  | H20 | Phang Nga | Human | EF200012 | Sukmee et al., 2008 |
|  | H21 | Brazil | Horse | OP328766 | Mendes et al., 2023 |
|  | H22 | Germany | Horse | GQ281278  GQ281279  GQ281280  GQ281281 | Muller et al., 2009 |
|  |  | Switzerland | Cow | GQ281282 | Lobsiger et al., 2010 |
|  | H23 | Lamphun | *Culicoides mahasarakhamense* | MW652868  MW652869  MW652870 | Sunantaraporn et al., 2021 |
|  | H24 | Turkey | *Phlebotomus major* | OQ863738 | Yildirim et al., 2023 (Unpublished) |
|  | H25 | Trang | Human | KY982647 | Manomat et al., 2017 |
|  | H26 | Trang | Human | KY982648 | Manomat et al., 2017 |
|  | H27 | Songkhla | *Culicoides peregrinus* | OP698054 | Songumpai et al., 2022 |
|  | H28 | Songkhla | *Culicoides peregrinus* | OP698057 | Songumpai et al., 2022 |
|  | H29 | Trang | Human | KY982644 | Manomat et al., 2017 |
|  | H30 | Trang | Human | KY982643  KY982649 | Manomat et al., 2017 |
|  | H31 | Songkhla | *Culicoides oxystoma* | OP698058 | Songumpai et al., 2022 |
|  | H32 | Songkhla | *Culicoides oxystoma* | OP698061 | Songumpai et al., 2022 |

**Table S1**. The information on 167 *ITS1* sequences of *L.* *martiniquensis* and *L. orientalis* isolates in humans, *Culicoides* biting midges, and animal hosts from Thailand and other geographical regions. (Continued)

| *Leishmania* species | Haplotype | Country/Provinces | Host | Accession numbers | References |
| --- | --- | --- | --- | --- | --- |
| *L. orientalis* | H33 | Chiang Rai | *Culicoides* spp. | PP586234 (4) | Ampol et al., 2024 |
|  |  | Nan | Human | MG731230 | Jariyapan et al., 2018 |
|  |  | Trang | Human | KY982662  KY982664  KY982665  KY982667  KY982668  KY982670  KY982673  KY982675 | Manomat et al., 2017 |
|  |  |  |  | MH807724 | Pandey et al., 2018 |
|  | H34 | Chiang Rai | *Culicoides* (*Trithecoides*) sp. | PP586237 | Ampol et al., 2024 |
|  | H35 | Chiang Rai | *Culicoides mahasarakhamense* | PP586239 | Ampol et al., 2024 |
|  | H36 | Chiang Rai | *Culicoides guttifer* | PP586248 | Ampol et al., 2024 |
|  | H37 | Chiang Rai | *Culicoides jacobsoni* | PP586249 | Ampol et al., 2024 |
|  | H38 | Chiang Rai | *Culicoides mahasarakhamense* | PP586250 | Ampol et al., 2024 |
|  | H39 | Chiang Rai | *Culicoides mahasarakhamense* | PP586246 | Ampol et al., 2024 |
|  | H40 | Trang | Human | KY982671 | Manomat et al., 2017 |
|  | H41 | Lampang | *Culicoides oxystoma* | OR917779 | This study |
|  | H42 | Chiang Rai | *Culicoides* (*Trithecoides*) sp. | PP586235 | Ampol et al., 2024 |
|  | H43 | Trang | Human | KY982677 | Manomat et al., 2017 |
|  | H44 | Lampang | *Culicoides oxystoma* | OR917780  OR917781 | This study |
|  |  | Nakhon Si Thammarat | Human | ON303842 | Anugulruengkitt et al., 2022 |
|  |  | Trang | Human | JX195640 | Leelayoova et al., 2013 |
|  | H45 | Trang | Human | KY982666 | Manomat et al., 2017 |
|  | H46 | Trang | Human | KY982658 | Manomat et al., 2017 |
|  | H47 | Chiang Rai | *Culicoides orientalis* | PP586240 | Ampol et al., 2024 |
|  | H48 | Chiang Rai | *Culicoides guttifer* | PP586245 | Ampol et al., 2024 |
|  | H49 | Chiang Rai | *Culicoides guttifer* | PP586241 (3) | Ampol et al., 2024 |
|  |  | Trang | Human | KY982674 | Manomat et al., 2017 |
|  |  | Songkhla | *Culicoides peregrinus* | OP698056 | Songumpai et al., 2022 |
|  | H50 | Trang | Human | KY982669 | Manomat et al., 2017 |
|  | H51 | Chiang Rai | *Culicoides mahasarakhamense* | PP586242 | Ampol et al., 2024 |
|  | H52 | Chiang Rai | *Culicoides mahasarakhamense* | PP586243 | Ampol et al., 2024 |
|  | H53 | Chiang Rai | *Culicoides oxystoma* | PP586244 | Ampol et al., 2024 |
|  | H54 | Trang | Human | KY982663  KY982676 | Manomat et al., 2017 |
|  | H55 | Trang | Human | KY982672 | Manomat et al., 2017 |
|  | H56 | Trang | Human | KY982659  KY982660  KY982661 | Manomat et al., 2017 |
|  | H57 | Chiang Rai | *Culicoides mahasarakhamense* | PP586247 | Ampol et al., 2024 |
|  | H58 | Chiang Rai | *Culicoides* (*Trithecoides*) sp. | PP586236 | Ampol et al., 2024 |
|  | H59 | Chiang Rai | *Culicoides* (*Trithecoides*) sp. | PP586238 | Ampol et al., 2024 |

**References**

Ampol R, Somwang P, Khositharattanakool P, Promrangsee C, Pataradool T, Tepboonreung P, et al. Nanopore-Based Surveillance of *Leishmania* Parasites in *Culicoides* Latrielle (Diptera: Ceratopogonidae) Caught from the Affected Community and Tham Phra Cave in Chiang Rai Province, the Endemic Area of Leishmaniasis in Northern Thailand. Insects. 2024;15:327.

Anugulruengkitt S, Songtaweesin WN, Thepnarong N, Tangthanapalakul A, Sitthisan M, Chatproedprai S, et al. Case Report: Simple Nodular Cutaneous Leishmaniasis Caused by Autochthonous *Leishmania* (*Mundinia*) *orientalis* in an 18-Month-Old Girl: The First Pediatric Case in Thailand and Literature Review. Am J Trop Med Hyg. 2023;108:44-50.

Chiewchanvit S, Tovanabutra N, Jariyapan N, Bates MD, Mahanupab P, Chuamanochan M, et al. Chronic generalized fibrotic skin lesions from disseminated leishmaniasis caused by *Leishmania martiniquensis* in two patients from northern Thailand infected with HIV. Br J Dermatol, 2015;173:663–670.

Chusri S, Hortiwakul T, Silpapojakul K, Siriyasatien P. Consecutive cutaneous and visceral leishmaniasis manifestations involving a novel *Leishmania* species in two HIV patients in Thailand. Am J Trop Med Hyg. 2012;87:76-80.

Jariyapan N, Daroontum T, Jaiwong K, Chanmol W, Intakhan N, Sor-Suwan S, et al. *Leishmania* (*Mundinia*) *orientalis* n. sp. (Trypanosomatidae), a parasite from Thailand responsible for localised cutaneous leishmaniasis. Parasit Vectors. 2018;11:351.

Kaewmee S, Mano C, Phanitchakun T, Ampol R, Yasanga T, Pattanawong U, et al. Natural infection with *Leishmania* (*Mundinia*) *martiniquensis* supports *Culicoides peregrinus* (Diptera: Ceratopogonidae) as a potential vector of leishmaniasis and characterization of a *Crithidia* sp. isolated from the midges. Front Microbiol. 2023;14:1235254.

Leelayoova S, Siripattanapipong S, Hitakarun A, Kato H, Tan-ariya P, Siriyasatien P, et al. Multilocus characterization and phylogenetic analysis of *Leishmania siamensis* isolated from autochthonous visceral leishmaniasis cases, southern Thailand. BMC Microbiol. 2013;13:60.

Lobsiger L, Müller N, Schweizer T, Frey CF, Wiederkehr D, Zumkehr B, et al. An autochthonous case of cutaneous bovine leishmaniasis in Switzerland. Vet Parasitol. 2010;169:408-414.

Manomat J, Leelayoova S, Bualert L, Tan-Ariya P, Siripattanapipong S, Mungthin M, et al. Prevalence and risk factors associated with *Leishmania* infection in Trang Province, southern Thailand. PLoS Negl Trop Dis. 2017;11:e0006095.

Mendes Junior AAV, Filgueira CPB, Miranda LFC, de Almeida AB, Cantanhêde LM, Fagundes A et al. First report of *Leishmania* (*Mundinia*) *martiniquensis* in South American territory and confirmation of Leishbunyavirus infecting this parasite in a mare. Mem Inst Oswaldo Cruz. 2023:15:e220220.

Müller N, Welle M, Lobsiger L, Stoffel MH, Boghenbor KK, Hilbe M, et al. Occurrence of *Leishmania* sp. in cutaneous lesions of horses in Central Europe. Vet Parasitol. 2009;166:346-351.

Noppakun N, Kraivichian K, Siriyasatien P. Disseminated dermal leishmaniasis caused by *Leishmania siamensis* in a systemic steroid therapy patient. Am J Trop Med Hyg. 2014;91:869-870.

Pandey N, Siripattanapipong S, Leelayoova S, Manomat J, Mungthin M, Tan-Ariya P, et al. Detection of *Leishmania* DNA in saliva among patients with HIV/AIDS in Trang Province, southern Thailand. Acta Trop. 2018;185:294-300.

Pothirat T, Tantiworawit A, Chaiwarith R, Jariyapan N, Wannasan A, Siriyasatien P, et al. First isolation of *Leishmania* from Northern Thailand: case report, identification as *Leishmania martiniquensis* and phylogenetic position within the *Leishmania enriettii* complex. PLoS Negl Trop Dis. 2014;8:e3339.

Reuss SM, Dunbar MD, Calderwood Mays MB, Owen JL, Mallicote MF, Archer LL, et al. Autochthonous *Leishmania siamensis* in horse, Florida, USA. Emerg Infect Dis. 2012;18:1545-7.

Songumpai N, Promrangsee C, Noopetch P, Siriyasatien P, Preativatanyou K. First Evidence of Co-Circulation of Emerging *Leishmania martiniquensis*, *Leishmania orientalis*, and *Crithidia* sp. in *Culicoides* Biting Midges (Diptera: Ceratopogonidae), the Putative Vectors for Autochthonous Transmission in Southern Thailand. Trop Med Infect Dis. 2022;7:379.

Srivarasat S, Brownell N, Siriyasatien P, Noppakun N, Asawanonda P, Rattanakorn K, et al. Case Report: Autochthonous Disseminated Cutaneous, Mucocutaneous, and Visceral Leishmaniasis Caused by *Leishmania martiniquensis* in a Patient with HIV/AIDS from Northern Thailand and Literature Review. Am J Trop Med Hyg. 2022;107:1196-202.

Suankratay C, Suwanpimolkul G, Wilde H, Siriyasatien P. Autochthonous visceral leishmaniasis in a human immunodeficiency virus (HIV)-infected patient: the first in thailand and review of the literature. Am J Trop Med Hyg. 2010;82:4-8.

Sukmee T, Siripattanapipong S, Mungthin M, Worapong J, Rangsin R, Samung Y, et al. A suspected new species of *Leishmania*, the causative agent of visceral leishmaniasis in a Thai patient. Int J Parasitol. 2008;38:617–622.

Sunantaraporn S, Thepparat A, Phumee A, Sor-Suwan S, Boonserm R, Bellis G, et al. *Culicoides* Latreille (Diptera: Ceratopogonidae) as potential vectors for *Leishmania martiniquensis* and *Trypanosoma* sp. in northern Thailand. PLoS Negl Trop Dis. 2021;15:e0010014.
